# Entity-enhanced BERT for medical specialty prediction based on clinical questionnaire data

**DOI:** 10.1371/journal.pone.0317795

**Published:** 2025-01-30

**Authors:** Soyeon Lee, Ye Ji Han, Hyun Joon Park, Byung Hoon Lee, DaHee Son, SoYeon Kim, HyeonJong Yang, TaeJun Han, EunSun Kim, Sung Won Han

**Affiliations:** 1 School of Industrial and Management Engineering, Korea University, Seongbuk-gu, Seoul, Republic of Korea; 2 People’s Health Co., Ltd., 403, BT-IT Convergence Center, Seongbuk-gu, Seoul, Republic of Korea; Cairo University, EGYPT

## Abstract

A medical specialty prediction system for remote diagnosis can reduce the unexpected costs incurred by first-visit patients who visit the wrong hospital department for their symptoms. To develop medical specialty prediction systems, several researchers have explored clinical predictive models using real medical text data. Medical text data include large amounts of information regarding patients, which increases the sequence length. Hence, a few studies have attempted to extract entities from the text as concise features and provide domain-specific knowledge for clinical text classification. However, it is still insufficient to inject them into the model effectively. Thus, we propose Entity-enhanced BERT (E-BERT), which utilizes the structural attributes of BERT for medical specialty prediction. E-BERT has an entity embedding layer and entity-aware attention to inject domain-specific knowledge and focus on relationships between medical-related entities within the sequences. Experimental results on clinical questionnaire data demonstrate the superiority of E-BERT over the other benchmark models, regardless of the input sequence length. Moreover, the visualization results for the effects of entity-aware attention prove that E-BERT effectively incorporate domain-specific knowledge and other information, enabling the capture of contextual information in the text. Finally, the robustness and applicability of the proposed method is explored by applying it to other Pre-trained Language Models. These effective medical specialty predictive model can provide practical information to first-visit patients, resulting in streamlining the diagnostic process and improving the quality of medical consultations.

## Introduction

The COVID-19 pandemic has led to the rapid development of non-face-to-face online systems in several fields, such as education, commerce, and entertainment. They have changed our daily lives in various ways, and a lot of people prefer these systems since they are free from space and time constraints. As the number of patients increases due to the coronavirus, extensive studies have been conducted to utilize remote diagnosis systems for improving the efficiency of the diagnostics process, even in the medical field [1–3].

Progress in deep learning has prompted researchers to apply artificial intelligence (AI) to remote diagnostic systems for doctors and patients. Specifically, a medical specialty prediction system based on clinical text classification can reduce the cost incurred because of the mismatch between the symptoms of first-visit patients and the recommended departments in the clinical process. Having patients visit the correct department is important for minimizing the unnecessary waste of resources used by doctors and hospital staff, who must also manage other medical appointments. Furthermore, this predictive system can enhance the quality of communication between doctors and patients, resulting in precise diagnoses and higher patient satisfaction [4].

For clinical predictive models, several researchers have utilized real medical text data, such as electronic medical records (EMRs), electronic health records (EHRs) [5, 6], and descriptions of symptoms scraped from medical websites [7, 8]. Real medical text data usually contain various aspects of patient information. For instance, EMRs and EHRs are large-scale, unstructured medical datasets that include information about a patient’s diagnostic history, surgical records, laboratory results, and other personal data. Descriptions scraped from websites, which are often written by non-professionals, may contain inaccurate or ambiguous information with diverse contents. Therefore, it is important to extract concise features and provide domain-specific knowledge to capture the contextual information for clinical text classification.

Several studies have utilized medical-related entities extracted from input text to represent its medical knowledge. However, they do not sufficiently reflect the importance of the entities and their relationships within a text. Mugisha and Paik [9] used only entities extracted from medical notes as one of the pipelines for disease prediction. However, relationships between entities and between an entity and the entire text cannot be reflected from this information. In addition, this method may result in the loss of valuable information from other sentences. He et al. [10] proposed KG-MTT-BERT, which extends the BERT framework and integrates the medical knowledge graphs based on entities for multi-type text classification. Since they built two independent frameworks, one for the input text and the other for the entities, the relationships between the input text and entities could not be reflected directly, and the model has high complexity. Therefore, more sophisticated text classification methods that can effectively reflect domain-specific knowledge in medical texts are required.

To overcome the above limitations, in this study, we propose an Entity-enhanced BERT (E-BERT), which is a single framework for medical specialty prediction. Recently, with the emergence of the Transformer [11], the use of Pre-trained Language Models (PLMs) have become increasingly popular for clinical text classification. In particular, Bidirectional Encoder Representations from Transformers (BERT) [12] has shown promising results in various studies [7–9]. Thus, we adopted the BERT framework to take advantage of LM architectures. E-BERT utilizes entity information in the embedding block and self-attention modules, thereby enabling the dual integration of domain-specific knowledge. In the embedding block, we introduce an entity embedding layer to inject domain-specific knowledge based on entities related symptoms, pain location, and diseases. Moreover, in the standard self-attention module of the BERT encoder, we introduce an entity-aware attention including the gate layer to emphasize the relationships between entities. By combining the two proposed modules, E-BERT enables the generation of enriched representations of medical texts. E-BERT was evaluated on the patient questionnaire data acquired from a university hospital in South Korea.

The contributions of this study are as follows:

1. We propose a single framework E-BERT to create a domain-specific contextual representation for medical text. E-BERT comprises advanced embedding block and self-attention modules to represent the meaning and relationships of entity information for medical specialty prediction.
2. We demonstrate that E-BERT outperforms other deep learning approaches for clinical text classification. The comparison of performance based on sequence length shows the superiority of E-BERT for dealing with long sequences, suggesting it is suitable for modeling medical text.
3. We prove that the diverse entity information and relationships between entities enhance the capture of contextual information through the visualization results.
4. The robustness of the proposed modules is demonstrated on two different PLMs with remarkable improvements on predictive performance.

## Related works

### Clinical text classification

Clinical text classification is a primary task in medical Natural Language Processing (NLP). It has been developed in several years, starting with statistical or machine learning approaches. Linear Regression (LR) [13], Naïve Bayes (NB) [14], and Support Vector Machines (SVMs) [15] are widely used to classify clinical texts. Specifically, Pagad et al. [16] performed comparative experiments based on various machine learning algorithms such as Random Forest, XGBoost, AdaBoost, and CatBoost. The superiority of deep learning models has been observed in clinical text classification owing to their versatility across different domains. Models based on one-dimensional Convolutional Neural Network (CNN) [17] and Recurrent Neural Network (RNN) [18] have been suggested for clinical text classification and have proved to outperform other comparative models. Furthermore, hybrid models combined with CNN, RNN-based models, and other algorithms have been proposed for effective feature integration and a robust architecture [8, 19, 20]. However, they have the fundamental limitation of enriched word representation owing to static word embeddings; for example, TF-IDF, Word2Vec [21], GloVe [22], and FastText [23], cannot reflect the context of a sentence [24]. The emergence of LMs has addressed this limitation and allowed contextualized word representations by considering the relationships between words in sentences. Particularly, BERT, one of the LMs, has brought substantial transformations in the clinical text classification. Kim et al. [7] proposed a medical specialty prediction model in which a domain-specific BERT was fine-tuned using a medical question-answer (QA) dataset. This model showed higher performance compared with other deep learning models such as CNN and Long Short-Term Memory (LSTM). Mao et al. [8] developed a hybrid model, HyM, based on BERT to classify medical symptom descriptions. BERT extracts attention vectors to weigh the feature vectors extracted from the other modules of HyM. For disease classification, Mugisha and Paik [9] used BERT to create a modeling pipeline for outcome prediction based on the clinical text notes of patients with pneumonia.

### Bidirectional encoder representations from transformers

In recent years, with the emergence of Transformer [11], BERT [12], which consists of multi-layer Transformer encoder, has been widely employed. BERT is a PLM trained on large amounts of text data in an unsupervised manner before being fine-tuned for downstream tasks. BERT adopts two tasks for pre-training: Masked LM (MLM) and Next Sentence Prediction (NSP). In the MLM, a certain percentage of tokens in a sentence is randomly masked, and the model is trained to predict the masked tokens based on the neighboring context. This allows the model to learn deep bidirectional representations, unlike other PLMs, such as Embeddings from Language Models [25] and Generative Pre-trained Transformer [26]. NSP involves predicting whether two sentences will follow each other in a given text. This allows the model to learn semantic relationships and understand the context between sentences. BERT has achieved state-of-the-art performance in various tasks, such as text classification, named entity recognition, and question and answering.

To take advantage of the structural superiority and flexibility of adapting to tasks, several researchers have developed task-specific BERT models, regardless of the domain. In the medical field, Radford et al. [26] improved BERT by employing additive attention in the last encoder layer for biomedical relationship extraction. Rasmy et al. [27] suggested Med-BERT, in which an ICD code and serialized embedding layer are added to the BERT embedding block for disease prediction. These approaches demonstrate the adaptability of BERT for various tasks within the medical field, showcasing the potential of tailored BERT-based models to enhance performance in specific medical tasks. In this study, we proposed E-BERT, by developing a tailored embedding block and self-attention module for medical specialty classification, utilizing the advantages of BERT.

### Entity-aware models

Since the entity information extracted from text provides valuable and concise features, its importance has been emphasized in various NLP tasks. To effectively utilize entity information, several researchers have developed entity-aware models that incorporate this information into models for specific tasks. Lee et al. [28] proposed a LSTM-based entity-aware attention model that applies self-attention in entity pairs with their latent types to focus on important semantic information for relation classification. Li et al. [29] applied the attention mechanism with entity-aware embedding to piecewise CNN. They constructed word embedding with an entity pair, resulting in the injection of entity information into the input representation. Furthermore, Yamada et al. [30] introduced LUKE, a PLM with a novel pre-training task extended the conventional MLM to learn entity representations, for entity-related tasks. For downstream tasks, they used entity-aware self-attention with three different query matrices applied differently to tokens, according to the possible pairs between token types.

In the medical field, entity-aware models have been developed to represent professional knowledge of complicated medical texts. For medical dialogue generation, entity information is combined with input dialogue in the encoder stage [31, 32]. Xiong et al. [31] proposed Entity-enhanced Dialogue Generation (EDG) model, which is an encoder-decoder framework based on Gated Recurrent Unit (GRU) [33] with entity-category embedding. EDG encodes historical dialogues as well as the corresponding entity information separately and fuses them using an attention mechanism. Li et al. [32] proposed the encoding fusion mechanism, which averages dialogue and entity features extracted by cross-attention with the previous token separately. For clinical text classification, He et al. [10] incorporated medical text and entity features that were extracted from independent frameworks such as BERT and Transformer with a pooling strategy. They were then concatenated and classified into diagnosis-related groups.

Previous studies on clinical models have achieved performance gains by incorporating medical knowledge derived from the entity information. However, these methods do not effectively consider the relationships between entities within a text because of their independent frameworks. This may potentially prevent the model from creating an enriched representation reflecting entity information. Furthermore, independent frameworks for text and entities result in high model complexity. To overcome these limitations, we propose an entity embedding layer and entity-aware attention which can be incorporated into the architecture of PLMs. These can improve the quality of the representations of entity-specific contexts in medical texts.

## Methodology

**Fig 1:**
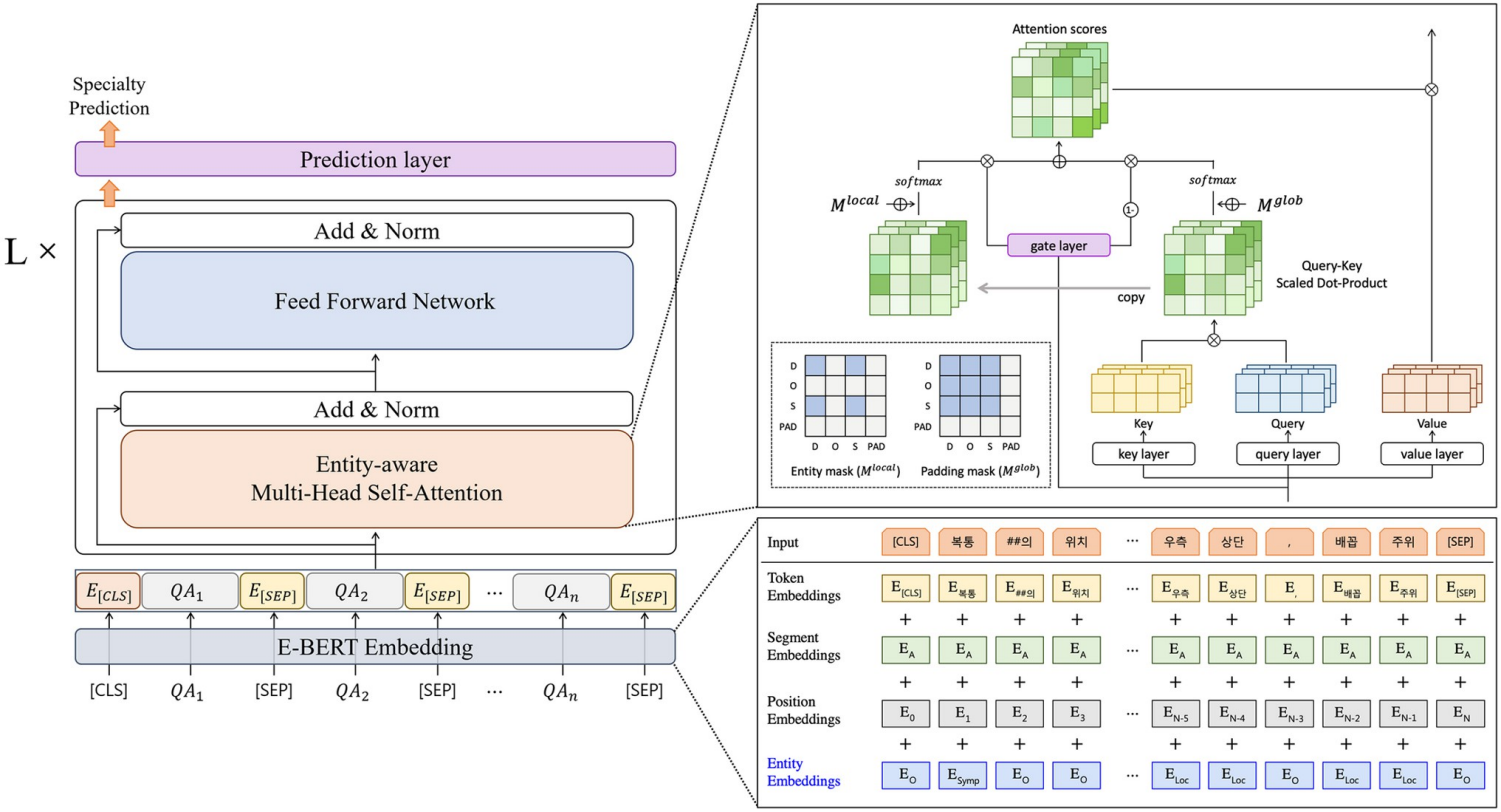
Overall framework for our proposed method. The architecture of E-BERT (left), and the detailed implementation of the E-BERT embedding block and entity-aware multi-head self-attention module (right).

In this section, we introduce the overall framework for predicting medical specialties in detail. Medical Named Entity Recognition, which is employed to extract concise and informative features (entities) related to symptoms, pain location, and disease in the input sequences, is represented. E-BERT, which is composed of four modules for medical specialty prediction, is introduced. As shown in Fig 1, E-BERT was designed to effectively incorporate entity information into the BERT architecture. The key components of E-BERT—the entity embedding layer and the entity-aware attention module—are presented, respectively.

### Dataset

This study used questionnaire data provided by Korea University Anam Hospital, which was approved by Korea University Medicine Institutional Review Board (IRB). The approval number for this data is 2021AN0328. The questionnaire dataset consisted of 27,037 QA pairs with 56,759 sentences from 1,788 participants. It was collected for four types of medical specialties related to abdominal symptoms, including gastroenterology: upper gastrointestinal, lower gastroenterology, pancreaticobiliary part, and colon and rectal surgery. The data mainly consisted of not only key information, including symptoms, location of pain, medical history, and underlying diseases, but also additional information, including age, sex, body measurements, family history, and lifestyle factors. Text preprocessing was conducted to correct typographical or grammatical errors by utilizing *py-hanspell*, a Python package that checks korean spelling.

The data was accessed for research purposes on December, 12, 2023. We did not have access to information that could identify individual participants during or after data collection. All data used in the study were fully anonymized before the researchers accessed them.

### Medical named entity recognition

**Fig 2:**
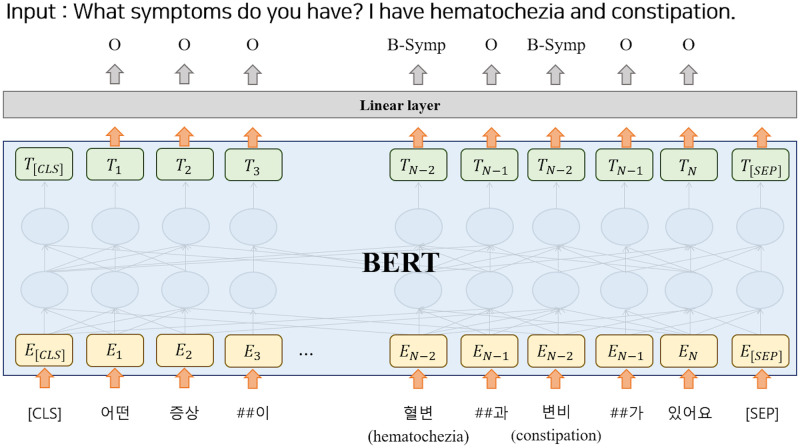
Medical NER model for sequence-level prediction.

Named Entity Recognition (NER), which identifies text spans and categorizes them into one of the classes based on a pre-defined set, is the main research field in NLP. This is crucial for language understanding applications in various domains. In the medical domain, several researchers have studied NER systems to extract keywords from raw medical text notes [9, 34, 35], and have shown that NER systems are effective and practical methods for various tasks. In this study, we trained a Medical NER model to extract key entities from input sequences and used the results to classify medical specialties. Table 1 lists the entity distributions in the dataset. The model uses three types of entities—disease, location, and symptom—to recognize each related token. Korean Medical BERT (KM-BERT) [36] is a domain-specific pre-trained language model trained on Korean medical textual data. It was fine-tuned to the clinical questionnaire data labeled at the token-level according to the BIO tagging method with experts, as shown in Fig 2. The Medical NER model was trained at the sequence-level and was used to predict the positions of entities by classifying each token into one of the entity types in a sequence. Finally, we incorporated information on the position of an entity into the BERT architecture to enhance the performance of medical specialty prediction.

**Table 1:** Medical entity distribution in the dataset.

Entity	Examples	Count
Symptom	stomachache, headache, indigestion	10,983
Location	thorax, abdomen, esophagus	8,313
Disease	ulcerative colitis, diabete mellitus	850

### Entity-enhanced BERT for medical specialty prediction

#### Model input

The clinical questionnaire data comprised several question-answer pairs for each participant. To construct the model input, QA pairs are sequentially concatenated into a single sequence. The input sequence is given as follows:

$$\begin{array}{c} \left\{\left[\text{CLS}\right]\;{QA}_{1}\;\left[\text{SEP}\right]\;{QA}_{2}\;\cdots \;\left[\text{SEP}\right]\;{QA}_{n}\;\left[\text{SEP}\right]\right\} \end{array}$$ (1)

where [CLS] and [SEP] are the special tokens used by BERT to denote the beginning and separation of sentences, respectively. The output of the [CLS] token is used to predict the specialty in the prediction layer. The input sequence is tokenized into subwords, resulting in $X_{token}=\left\{x_{1}^{t},\;x_{2}^{t},\;\cdots ,\;x_{n}^{t}\right\}$. In addition, we input a sequence of entity types corresponding to each token into the model, denoted as $X_{entity}=\left\{x_{1}^{e},\;x_{2}^{e},\;\cdots ,\;x_{n}^{e}\right\}$, which represents the output from the Medical NER.

#### Embedding block

The BERT embedding block contains a token embedding layer, position embedding layer, and segment embedding layer. The representations for each token are created by summing each embedding output. Token embeddings are word vectors to which tokens are mapped and represent the semantic information of individual words. Position embeddings are used to inject information regarding the order of each token in the sequences. Finally, segment embeddings are used to separate the different sentences in the input text. These three types of embeddings generate contextualized representations of the input sequences.

In this study, based on the design of embedding block, we add an entity embedding layer to inject domain-specific information to each token in QA sequences. The entity embedding layer, which is a linear projection layer, maps the entity type of each token to an embedding vector. The final embedding vector fed into the E-BERT encoder is defined as follows:

$$\begin{array}{c} e_{w}=\;e_{w}^{token}+e_{w}^{pos}+e_{w}^{seg}+e_{w}^{entity},\\ entity\in \{\text{Symptom},\;\text{Location},\;\text{Disease},\;\text{Others}\} \end{array}$$ (2)

where $e_{w}^{entity}$ represents the embedding vector for the corresponding entity type of the token *w*, one of the symptoms, location, disease, or others (representing tokens that do not belong to any specific entity type). The final embedding vector is denoted by $e_{w}\in R^{n\times d}$, where *n* denotes the sequence length and *d* denotes the dimension of the hidden vector.

#### Encoder

The E-BERT is designed based on the BERT encoder [12], which consists of L identical layers. Each encoder layer contains Entity-aware Multi-Head Self-Attention (MHSA), Feed Forward Network, residual connection, and layer normalization.

The Entity-aware MHSA module is an extension of the standard MHSA to emphasize the relationships between medical-related entities in the input sequences. The standard MHSA learns the representation of each token in input sequences from diverse perspectives with multiple heads. Each head calculates the attention scores for different context words based on the query *Q*, key *K*, and value *V* which are obtained by projecting hidden vectors from the previous layer. The calculation of self-attention for a head *k*, where *k* = {1, 2, ⋯, *H*}, can be represented as follows:

$$\begin{array}{c} M_{ij}^{glob}=\{\begin{array}{c} \;\;\;0,\;if\;x_{i}^{t}\;and\;x_{j}^{t}\;are\;not\;\left[PAD\right]\\ -\infty ,\;\;otherwise \end{array}\\ S_{k}^{glob}=softmax\left(\frac{Q_{k}K_{k}^{⊺}}{\sqrt{d_{k}}}+M^{glob}\right) \end{array}$$ (3)

where a scaling factor $\frac{1}{\sqrt{d_{k}}}$ divided by the dimensions of the key *d*_*k*_, is used to normalize the dot-product attention matrix $Q_{k}K_{k}^{⊺}$. Furthermore, the mask matrix *M*^*glob*^ is used to guide the flow of attention towards relevant positions by masking out unnecessary tokens (e.g., [PAD] tokens) in the input sentences. In this global attention mechanism, each token interacts with all the other tokens, resulting in a similarity matrix $S_{k}^{glob}$.

In addition, for the model to learn the relationships between medical-related entities in the entire input sequence, an entity-aware attention mechanism is adopted. This mechanism was inspired by [37], which overcame the limitation of window-based local attention restrained the attention scope within a linear span. The entity-aware attention mechanism allows attention to be paid only to medical-related tokens. If tokens *x*_*i*_ and *x*_*j*_ belong to medically relevant entity type E, where *E* = {*Symptom*, *Location*, *Disease*}, they can attend to each other. The local mask matrix *M*^*local*^ is added to the dot-product attention matrix $Q_{k}K_{k}^{⊺}$. The similarity matrix for the entity-aware attention mechanism is expressed as follows:

$$\begin{array}{c} M_{ij}^{local}=\{\begin{array}{c} \;\;\;0,\;if\;x_{i}^{e}\;and\;x_{j}^{e}\;\in E\\ -\infty ,\;\;otherwise \end{array}\\ S_{k}^{local}=softmax\left(\frac{Q_{k}K_{k}^{⊺}}{\sqrt{d_{k}}}+M^{local}\right) \end{array}$$ (4)

A gate mechanism is used to aggregate two types of similarity matrices: a global attention matrix *S*^*global*^ and an entity-aware attention matrix *S*^*local*^, as shown in Fig 1. It calculates the importance score of each token and weighs the attention score based on its value. The gate value *g* of all the tokens and the aggregated attention scores *O*_*k*_ are calculated as follows:

$$\begin{array}{c} g=\sigma (W_{g}^{⊺}h+b_{g})\\ O_{k}=\left(gS_{k}^{local}+\left(1-g\right)S_{k}^{glob}\right)V_{k} \end{array}$$ (5)

where *σ* denotes the sigmoid function; *h* denotes the hidden states from the previous layer; $W_{g}\in R^{d\times 1}$ denotes a linear transformation with a randomly initialized weight matrix; and *b*_*g*_ denotes a bias.

The representations for all heads are concatenated and updated using a linear transformation $W\in R^{d\times d}$ and a bias *b*:

$$\begin{array}{c} O=W^{⊺}\left(\left[O_{1};\;\cdots \;;O_{H}\right)\right])+b \end{array}$$ (6)

The Feed Forward Network module provides the final representation of each encoder layer. It contains two linear transformations and an activation function for the Gaussian error linear unit (GELU) [38]. The calculations for this module are formulated as follows:

$$\begin{array}{c} FFN\left(x\right)=W_{2}^{⊺}\left(GELU\left(W_{1}^{⊺}x+b_{1}\right)\right)+b_{2} \end{array}$$ (7)

where $W_{1}\in R^{d\times 4d}$ and $W_{2}\in R^{4d\times d}$ increase the dimensions of the hidden vectors by four times and then return them to extract a richer representation. The choice to increase the dimensions by four times follows a widely accepted practice in Transformer-based architectures, such as the original Transformer model [11] and BERT [12]. The final encoder output is obtained after passing through L identical layers.

#### Prediction layer

The final hidden state *h*_*cls*_ of the [CLS] token that represents the entire document is typically used for classification tasks. To predict a document, the hidden state *h*_*cls*_ passes through an additional fully connected layer with Softmax.

$$\begin{array}{c} p=softmax(W_{p}^{⊺}h_{cls}+b_{p}) \end{array}$$ (8)

where $W_{p}\in R^{d\times C}$ is a linear transformation that changes the dimension of $h_{cls}\in R^{d}$ to the number of classes, resulting in creating $p\in R^{C}$.

## Experimental setup

### Implementation details

The dataset was split into multiple folds using nested cross-validation (CV), which is an expanded technique of conventional cross-validation. Nested CV divides the data into outer folds, which are regarded as test sets, and inner folds, which are used to train the models. The inner folds for each outer fold are divided into a training set and a dev set again to select the best model parameters. This technique helps obtain an unbiased performance for each test set by preventing overfitting and allowing the evaluation of the model’s generalization for an unknown set. In this study, 5-fold outer cross-validation and 3-fold inner cross-validation were performed; that is, the total number of experiments for a single setting was 15.

The pre-trained BERT-base, which consists of 12 encoder layers with hidden sizes of 768 and 12 multi-heads, was used for the base model. For training, we set a batch size of 4 and an epoch of 30. Additionally, the Adam optimizer [39] with a weight decay of 1e-2 and a linear learning rate scheduler with a warm-up ratio of 0.1 were used to optimize the models. For the optimal convergence of each model, the learning rate was selected among {5e-4, 5e-6}. During training, the weights of the model were saved based on the validation macro F1 scores. All the experiments were performed using a single RTX 3090 GPU.

### Evaluation metrics

To evaluate the medical specialty predictions, a confusion matrix that provides detailed information about performance was adopted. The confusion matrix consists of four components: true positives (TP), true negatives (TN), false positives (FP), and false negatives (FN). Each component represents the number of instances classified correctly or incorrectly by the model. The Accuracy, Precision, Recall, and F1 score, which are derived from the confusion matrix, were adopted as evaluation metrics. Accuracy is the ratio of correct predictions to the number of entire data samples. Precision and Recall are the ratios of TPs out of the samples predicted by the model as positive and actual positive samples, respectively. The F1 score combines both Precision and Recall, providing a balanced evaluation metric that considers both FP and FN. To assign equal importance to all classes, the F1 score was computed by averaging the scores for each class and was called the macro F1 score. Each metric was formulated as follows:

$$\begin{array}{c} \begin{array}{cc} Accuracy & =\frac{TP+TN}{TP+TN+FP+FN}\\ Precision & =\frac{TP}{TP+FP}\\ Recall & =\frac{TP}{TP+FN}\\ F1\;score & =2\times \frac{Precision\times Recall}{Precision+Recall} \end{array} \end{array}$$ (9)

Finally, the Matthews Correlation Coefficient (MCC) was used as the evaluation metric. MCC calculates the correlation between the predictions and actual labels as follows:

$$\begin{array}{c} MCC=\frac{TP\times TN-FP\times FN}{\sqrt{\left(TP+FP)(TP+FN)(TN+FP)(TN+FN\right)}} \end{array}$$ (10)

### Benchmark models

The proposed method was compared with other benchmark models based on deep learning. Recent studies on clinical text classification have demonstrated the superiority of deep learning models, such as RNN-based, 1D-CNN, and hybrid models over statistical algorithms [8, 19, 20]. In addition, PLMs, which are Transformers-based models, have been shown to be effective for clinical text classification [7, 9]. In our comparison, we included bidirectional RNN, GRU, LSTM, TextCNN, and a hybird model, CNN+LSTM, as benchmark models. BERT, ELECTRA, and DistilBERT were added for comparison. In particular, BERT served as the baseline model to verify the effectiveness of the proposed method.

## Results and analysis

### Main results

All the experimental results reported in this study are the average values obtained from the five outer folds of the nested CV. For non-Transformers-based models, which are RNN-based and CNN-based models, Word2Vec [21] was utilized for extracting features from sequences. As shown in Table 2, E-BERT outperformed the other benchmark models across all evaluation metrics. In particular, compared to the base model BERT, the effectiveness of our proposed method was proven. Although E-BERT marginally outperformed BERT in terms of accuracy, it achieved a significant improvement in terms of other metrics that consider class imbalance. Improvements of approximately 2.1% in precision, 2.7% in recall, and 2.6% in macro F1 score were observed. MCC improved by 0.9%, indicating enhanced performance of the proposed method. These results highlight the effectiveness of E-BERT in achieving better classification performance on medical datasets with imbalanced class distributions. This suggests that injecting domain-specific knowledge and paying more attention to their relationships yield a more accurate prediction for clinical text classification.

**Table 2:** Comparison results of our proposed model with benchmark models.

Model	Accuracy	Precision	Recall	Macro F1	MCC
BiRNN	0.668	0.590	0.617	0.564	0.463
BiGRU	0.775	0.701	0.702	0.690	0.636
BiLSTM	0.668	0.611	0.595	0.558	0.460
TextCNN	0.813	0.729	0.741	0.724	0.696
CNN+LSTM	0.809	0.736	0.738	0.728	0.689
ELECTRA	0.765	0.726	0.672	0.648	0.621
DistilBERT	0.777	0.706	0.690	0.678	0.641
BERT (baseline)	0.830	0.756	0.746	0.734	0.726
**E-BERT (ours)**	**0.834**	**0.777**	**0.773**	**0.760**	**0.735**

**Fig 3:**
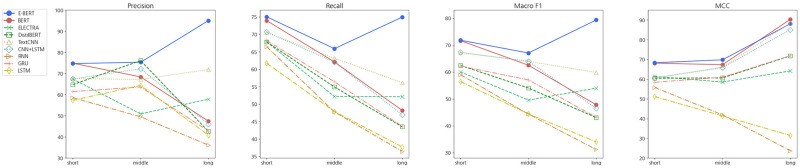
Comparison of prediction performance according to input sequence length in benchmark models and E-BERT. The samples were categorized as “short”, “middle”, and “long” based on the quartiles Q1 and Q3 of the sequence length distribution, considering their relative lengths in the dataset.

Furthermore, we compared the performance based on the input sequence length in benchmark models and E-BERT, as shown in Fig 3. As the sequence length increased, E-BERT consistently demonstrated superior performance, whereas most benchmark models exhibited performance degradation. In detail, E-BERT significantly outperformed BERT for long sequences across most metrics, with the exception of MCC. This indicates that E-BERT balances accurate predictions between classes regardless of the sequence length. This suggests that the incorporation of entity information enables the model to effectively capture contextual information in sequences, particularly when dealing with longer sequences.

### Ablation study

In this subsection, we discuss the ablation studies conducted to investigate the effects of the proposed method and medical-related entity types on performance.

#### Impact of components of E-BERT

The effects of the proposed modules were examined based on BERT, which is the base model for E-BERT. In Table 3, the embedding component refers to the additional entity embeddings in the BERT embedding block. The attention component represents the incorporation of entity-aware attention into the standard MHSA module of the BERT. The results in Table 3 indicate the importance of injecting domain-specific knowledge for each token into the proposed model. The embedding component generally slightly improved the performance in terms of most metrics, whereas the attention component degraded the performance across all evaluation metrics compared to BERT. However, E-BERT used both the embedding and attention components and exhibited a notably improved performance compared to BERT with the attention component. This result implies that in medical specialty prediction, relationships according to the meaning of each token should be reflected through the entity type.

**Table 3:** Ablation study of the two components of our proposed method.

Model	Pre.	Rec.	F1	MCC
BERT	0.756	0.746	0.734	0.726
w/ embedding	0.765	0.757	0.743	0.723
w/ attention	0.747	0.732	0.730	0.716
E-BERT	0.777	0.773	0.760	0.735

#### Impact of the entity types

Furthermore, the impacts of different entity types were explored using E-BERT. In this experiment, each entity type was individually removed, resulting in the consideration of each entity as part of the others type. The results show that the absence of information about each entity type reduces the overall performance compared with that of E-BERT, which contains information about all entity types, as demonstrated in Table 4. Particularly, Location type, which indicates the location of pain, caused a significant performance reduction in most metrics. This is because most of our data were collected from the gastroenterology department, which covers different parts of the body. This suggests that the interaction between information about the location of pain and symptoms/diseases is essential for predicting medical specialties. Therefore, by incorporating information about different entity types, E-BERT can understand the domain-specific context more precisely in clinical texts.

**Table 4:** Ablation study of the entity types in E-BERT.

Model	Pre.	Rec.	F1	MCC
E-BERT	0.777	0.773	0.760	0.735
w/o Symptom	0.754	0.778	0.749	0.725
w/o Location	0.760	0.763	0.742	0.720
w/o Disease	0.763	0.776	0.755	0.737

### Further experiments

In this subsection, additional experiments were conducted to explore the impact of entity-aware attention on different positions in the layers. Furthermore, to prove the robustness of the proposed method, it was applied to other PLMs, ELECTRA and DistilBERT.

#### Impact of entity-aware attention layers

In this experiment, entity-aware attention was applied to subsets of the E-BERT encoder layers selected from different positions. The 12 layers in the E-BERT encoder were divided into five parts, four of which consisted of consecutive layers and one consisted of non-consecutive layers, as shown in Table 5.

**Table 5:** Comparison results according to entity-aware attention layers.

Model	Layers	Pre.	Rec.	F1	MCC
E-BERT	1–3	0.763	0.766	0.749	0.730
	4–6	0.764	0.767	0.753	0.729
	7–9	**0.777**	0.773	**0.760**	**0.735**
	10–12	0.767	0.754	0.744	0.730
	4,8,12	0.764	**0.784**	0.755	0.737
	1–12	0.747	0.769	0.751	0.724

The comparison results are presented in Table 5. Interestingly, E-BERT exhibited lower performance when entity-aware attention was applied to all layers than when it was applied to only a few layers. This discrepancy suggests that emphasizing entity information in every layer may potentially interfere with the model’s ability to capture other relevant information. Thus, some layers, rather than all, are sufficient to reflect the relationships between entities. Specifically, the intermediate layers exhibited better performance than the early or later layers.

**Fig 4:**
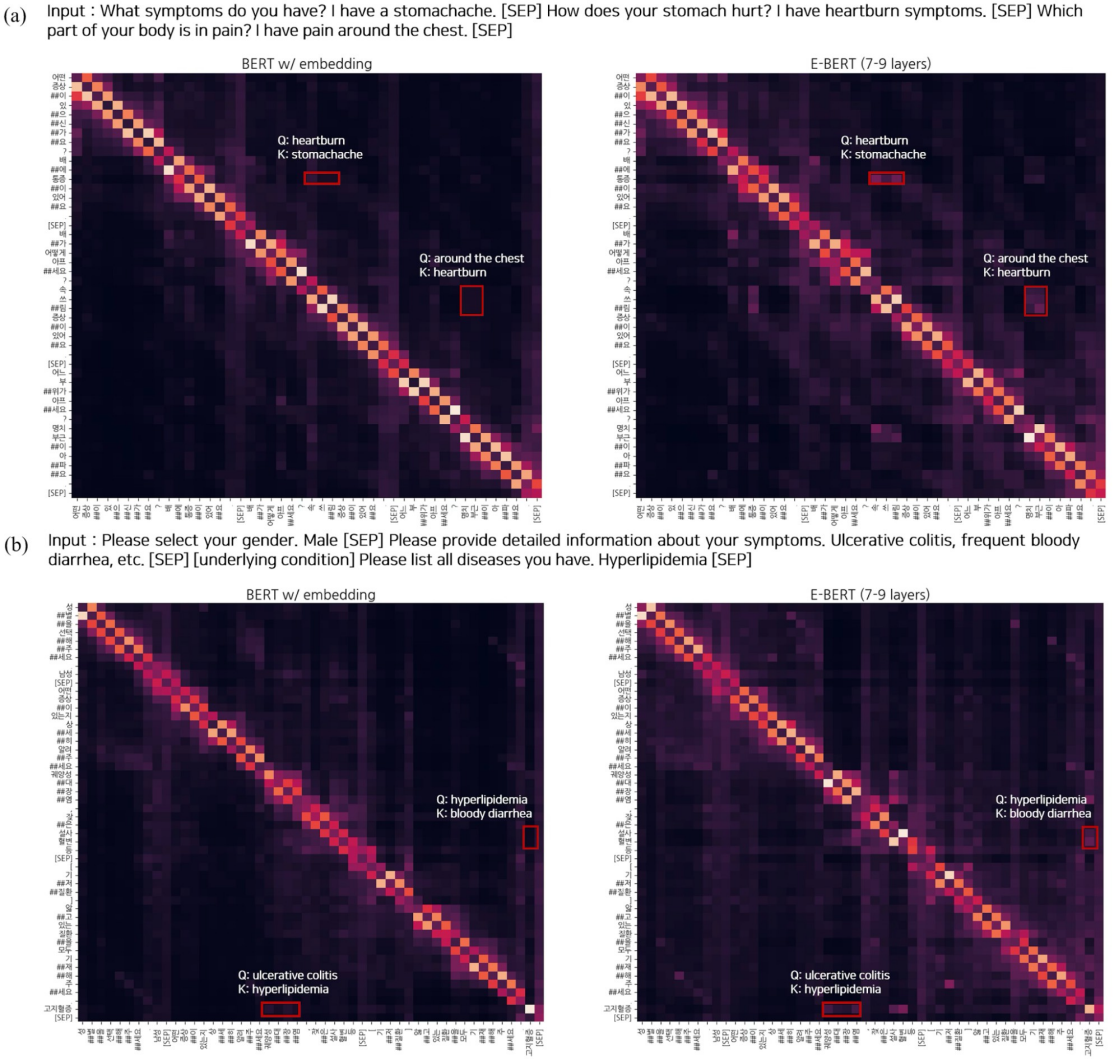
Visualization of the attention scores averaged across all heads and layers for two random samples in BERT (left) and E-BERT (right), respectively. In the case of E-BERT, we applied entity-aware attention to 7–9 layers of all the encoder layers. For the mapping between Korean tokens and their English translations, please refer to S1 Table.

To compare the attention distributions between BERT with entity embeddings and E-BERT, the attention maps for a portion of two random samples were visualized, as shown in Fig 4. The figure shows that E-BERT has a more global attention distribution, emphasizing medical-related tokens. In Fig 4(a), the tokens “명치 부근” (around the chest), which belong to the location type, pay attention to the tokens “속쓰림” (heartburn), which belong to symptom type. Similarly, the disease token “고지혈증” (hyperlipidemia) pays attention to the symptom tokens “혈변설사” (bloody diarrhea), as shown in Fig 4(b). This validates the effectiveness of the gate layers in entity-aware attention, which pays more attention to medical-related tokens and enhances their relationships. Hence, E-BERT can better capture contextual information based on domain-specific knowledge compared to BERT.

**Fig 5:**
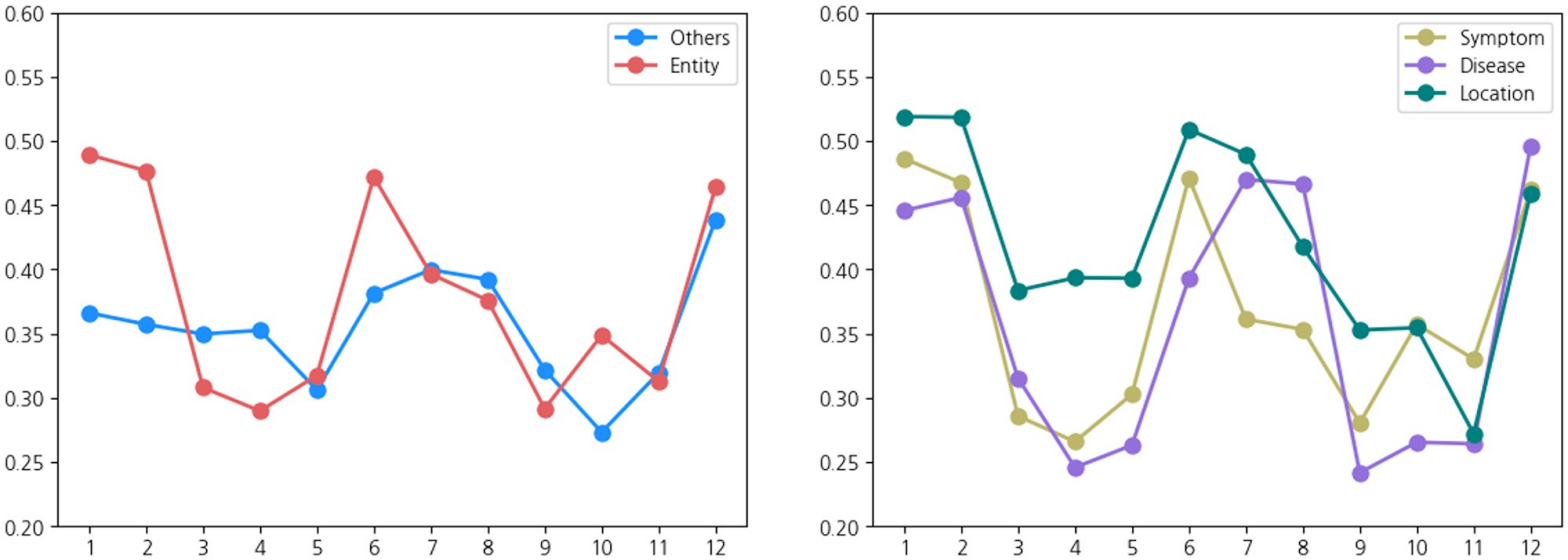
Gate values in each layer of E-BERT in which entity-aware attention is applied in all layers. The tokens of the medical-related entity type have larger gate values than other tokens in the early and middle layers (left). Specifically, the tokens of location type have the largest value in most layers (right).

The averaged gate values over the token types are visualized in Fig 5. E-BERT, which applies entity-aware attention to all the layers, was adopted to provide a comprehensive view of the operation of the entire model. E-BERT maintains a balance appositely between medical-related tokens and other tokens across all layers. Although medical-related tokens were activated more strongly than others in the early layers, there was an alternating pattern of increased activation across the layers. This indicates that the model initially focused on capturing domain-specific information and progressively integrated contextual information, resulting in a more comprehensive understanding of the input sequences. This observation highlights the ability of entity-aware attention to effectively incorporate medical knowledge and context, leading to enhanced performance. Moreover, the tokens of location type exhibit the overall dominant pattern of activated values across all layers compared to those of symptom and disease types. This suggests that E-BERT consider location information the most significantly for medical specialty prediction. Therefore, the importance of location type is reaffirmed, as also explicitly evidenced in Table 4.

#### Robustness of the proposed method

To prove the robustness and applicability of the proposed method, other PLMs, namely ELECTRA [40] and DistilBERT [41], which are variants of BERT, were adopted as base models.

ELECTRA, which shares the same architecture as BERT, employs a more efficient pre-training task called Replaced Token Detection (RTD) that overcomes the limitations of the MLM task of BERT based on a generator-discriminator framework. The generator-discriminator framework of RTD in ELECTRA involves training a generator to replace a subset of input tokens with plausible alternatives and a discriminator to distinguish between the original and replaced tokens. Through this framework, ELECTRA achieves improved performance and training efficiency compared with BERT. DistilBERT, an adeptly distilled variant of BERT, exhibits competitive performance with BERT while achieving a lower model capacity and accelerated inference speed. It comprises an embedding block with the segment embedding layer removed, followed by three Transformer encoder layers. These two different architectures of LM were adopted to apply the proposed method and evaluate its effectiveness and generalization across different PLMs.

As shown in Table 6, the proposed method yields significant performance enhancements in all evaluation metrics for the ELECTRA and DistilBERT frameworks. In the case of ELECTRA, specialties could be predicted more accurately by simply adding entity embeddings to inject medical knowledge, achieving performance competitive with that of E-ELECTRA. For DistilBERT, unlike ELECTRA, applying entity-aware attention without medical knowledge resulted in a significant improvement. These findings highlight the robustness and general applicability of the proposed method for leveraging entity information as domain-specific knowledge in diverse PLMs.

**Table 6:** The effectiveness of the proposed method based on ELECTRA and DistilBERT. In this experiment, entity-aware attention is naively applied to all encoder layers of each model.

Model	Accuracy	Precision	Recall	Macro F1	MCC
ELECTRA	0.765	0.726	0.672	0.648	0.621
w/ embedding	0.817	0.746	0.744	**0.726**	0.707
w/ attention	**0.821**	0.746	0.736	0.714	0.704
E-ELECTRA	0.817	**0.756**	**0.752**	0.725	**0.712**
DistilBERT	0.777	0.706	0.690	0.678	0.641
w/ embedding	0.812	**0.745**	0.712	0.717	0.690
w/ attention	0.821	0.740	**0.748**	0.728	**0.713**
E-DistilBERT	**0.823**	0.743	0.739	**0.731**	0.711

## Conclusion

This study proposed a single framework, E-BERT, to focus on entity information for medical specialty prediction. Specifically, the proposed modules, entity embedding layer and entity-aware attention, were built into the BERT architecture. The entity embedding layer injects domain-specific knowledge into the input representation. The entity-aware attention emphasizes the relationships between medical-related tokens. Experimental results show that E-BERT provides more balanced and accurate predictions than other benchmark models. In particular, E-BERT showed significant performance improvements as the length of the sequence increased. This suggests that the proposed modules contribute to enhance the performance by capturing contextual information regardless of the input sequence length. Furthermore, the visualization of the attention distribution and gate values shows that entity-aware attention effectively integrates medical knowledge and other information into the model. Finally, we proved the robustness of the proposed method by applying it to other PLMs, such as ELECTRA and DistilBERT, which have different model capacites or pretraining tasks. These clinical predictive models can help the first-visit patients avoid visiting hospital departments that are not suitable for treating their symptoms. Thus, they can reduce unexpected costs in the clinical diagnostic process and enhance the quality of medical consultations.

However, a limitation of our approach is its reliance on a two-stage process: initially extracting entity information using a separate NER system, followed by utilizing this information for medical specialty prediction. This separation may introduce cumulative errors and reduce overall efficiency. In future work, we aim to develop an end-to-end, one-stage model that simultaneously learns to recognize entities and predict medical specialties within a unified framework. Additionally, we plan to validate the effectiveness of this approach through real-time experimental analysis. We expect that this approach will not only improve performance but also simplify the real-time prediction process.

## Supporting information

S1 Table Mapping between English phrases and their corresponding Korean tokens. (PDF)
