# Supplementary material for: Entity-enhanced BERT for medical specialty prediction based on clinical questionnaire data
Source: PLoS One. 2025 Jan 30;20(1):e0317795. doi: 10.1371/journal.pone.0317795 (PMC11781728; doi:10.1371/journal.pone.0317795)
Supplement: S1 Table — (PDF) [file pone.0317795.s001.pdf]

Supporting Information

(a)

Korean:
어떤 증상이 있으신가요? 배에 통증이 있어요. [SEP] 배가 어떻게 아프세요? 속쓰림 증상이 있어요. [SEP] 어느 부위가 아프세요? 명치 부근이 아파요. [SEP]

English:
What symptoms do you have? I have a stomachache. [SEP] How does your stomach hurt? I have heartburn symptoms. [SEP] Which part of your body is in pain? I have pain around the chest. [SEP]

S1 TableA. Mapping between English phrases and their corresponding Korean tokens.

English	Korean
What	어떤
symptoms	증상, ##이
do you have?	있, ##으, ##신, ##가, ##요, ?
I have	있어요
a stomachache.	배, ##에, 통증, ##이, .
How does	어떻게
your stomach	배, ##가
hurt?	아프, ##세요, ?
I have	있어, ##요
heartburn	속, 쓰, ##림
symptoms.	증상, ##이, .
Which	어느
part of your body	부, ##위가
is in pain?	아프, ##세요, ?
I have pain	아, ##파, 요
around	부근, ##이
the chest.	명치, .

(b)

Korean:
성별을 선택해 주세요. 남성 [SEP] 어떤 증상이 있는지 상세히 알려주세요. 궤양성 대장염, 잦은 설사 혈변 등 [SEP] [기저질환] 앓고 있는 질환을 모두 기재해주세요. 고지혈증 [SEP]

English:
Please select your gender. Male [SEP] Please provide detailed information about your symptoms. Ulcerative colitis, frequent bloody diarrhea, etc. [SEP] [underlying condition] Please list all diseases you have. Hyperlipidemia [SEP]

S1 TableB. Mapping between English phrases and their corresponding Korean tokens.

English	Korean
Please	##주, ##세요
select	선택, ##해
your gender.	성, ##별, ##을, .
Male	남성
Please provide	알려, ##주, ##세요
detailed information	상, ##세, ##히
about your symptoms.	어떤, 증상, ##이, 있는지, .
Ulcerative	궤양성
colitis,	##대, ##장, ##염, ,
frequent	잦, ##은
bloody diarrhea,	설사, 혈변
etc.	등
[underlying condition]	[, 기, ##저, ##질환,]
Please	##주, ##세요
list	기, ##재, ##해
all	모두
disease	질환, ##을
you have.	앓, ##고, 있는, .

Hyperlipidemia	고지혈증
